# Extraction, Biochemical Characterization, and Health Effects of Native and Degraded Fucoidans from *Sargassum crispifolium*

**DOI:** 10.3390/polym14091812

**Published:** 2022-04-28

**Authors:** Wei-Cheng Hsiao, Yong-Han Hong, Yung-Hsiang Tsai, Yi-Chen Lee, Anil Kumar Patel, Hui-Ru Guo, Chia-Hung Kuo, Chun-Yung Huang

**Affiliations:** 1 Division of Gastroenterology (General Medicine), Department of Internal Medicine, Yuan’s General Hospital, No. 162, Cheng Kung 1st Rd., Lingya District, Kaohsiung City 80249, Taiwan; cheng_2034@yahoo.com.tw; 2 Department of Nutrition, I-Shou University (Yanchao Campus), No. 8, Yida Rd., Jiaosu Village, Yanchao District, Kaohsiung City 82445, Taiwan; yonghan@isu.edu.tw; 3 Department of Seafood Science, National Kaohsiung University of Science and Technology, No. 142, Haijhuan Rd., Nanzih District, Kaohsiung City 81157, Taiwan; yht@nkust.edu.tw (Y.-H.T.); lionlee@nkust.edu.tw (Y.-C.L.); kuo19921122@gmail.com (H.-R.G.); 4 Sustainable Environment Research Center, Department of Marine Environmental Engineering, National Kaohsiung University of Science and Technology, No. 142, Haijhuan Rd., Nanzih District, Kaohsiung City 81157, Taiwan; anilkpatel22@gmail.com

**Keywords:** antioxidant, cytokine, immunostimulant, fucoidan, Fourier transform infrared, *Sargassum crispifolium*

## Abstract

In the current investigation, a native crude fucoidan (Ex) was extracted from *Sargassum crispifolium*, pretreated by single-screw extrusion, and two degraded fucoidans, i.e., ExAh (degradation of Ex by ascorbic acid) and ExHp (degradation of Ex by hydrogen peroxide), were obtained. The extrusion pretreatment increased the extraction yield of fucoidan by approximately 1.73-fold as compared to the non-extruded sample. Among Ex, ExAh, and ExHp, their molecular weight and chemical compositions varied, but the structural features were similar. ExHp possessed the greatest antioxidant activities among the extracted fucoidans. According to the outcome, ExAh exhibited the maximum immune promoting effects via enhanced NO, TNF-α, IL-1β, IL-6, and IL-10 secretion. Thus, both ExHp and ExAh may potentially be used as an effective antioxidant and as immunostimulant agents, which could be of great value in the development of food and nutraceutical products.

## 1. Introduction

Brown algae have been observed to contain large amounts of various polysaccharides such as cellulose, fucoidans, and alginic acids [1]. Among these compounds, fucoidans appear to be the most prominent from a nutraceutical perspective. Fucoidans are a group of fucose-containing sulfated polysaccharides (FCSPs) consisting of mixtures of structurally related polysaccharides that have varying amounts of monosaccharide residues and non-carbohydrate components (predominantly sulfate and acetyl groups) [2]. These fucoidans have been demonstrated to possess several biological properties such as antioxidant, antibacterial, antivirus, anti-inflammatory, antiadipogenic, antitumor, anticoagulant, and antithrombotic, as well as neuroprotective effects [3,4,5,6,7]. Some studies have indicated that the molecular weight of fucoidan may play a crucial role in the biological actions of polysaccharides [8,9]. For instance, a low molecular weight (LMW) fucoidan has higher anticancer [8,9], anticoagulation, and antioxidative properties [10]. In addition, an LMW fucoidan is capable of enhancing revascularization of hindlimb ischemia in rats [11], promoting osteoblast proliferation in bone regeneration [12], and increasing formation of human endothelial cells [13]. Therefore, it is crucial to gain a better understanding of the production of LMW fucoidans and their health-promoting effects.

Reactive oxygen species (ROS) generally exist as superoxide anion (^•^O_2_^−^), hydroxyl radical (^•^OH), and hydrogen peroxide (H_2_O_2_). They are created as a consequence of normal cellular metabolism, but they can also be generated by exogenous factors. As such, they can be regarded as biomarkers of carcinogenesis, inflammation, and ischemia/reperfusion [14,15,16]. The vast majority of organisms possess antioxidant defense systems which can repair oxidative damage. Nonetheless, these systems cannot completely mitigate these deleterious effects. Moreover, certain synthetic antioxidants may constitute a potential health hazard for consumers [17]. Thus, there would be considerable demand from a variety of sectors, including the pharmaceutical and food industries, for potent, safe, and cheap antioxidants derived from naturally occurring compounds, especially ones that could deliver quantifiable health benefits.

Immunity has been defined as an organism’s ability to defend itself against foreign bodies and pathogenic microorganisms by identifying and destroying them [18]. Innate immunity is the first line of defense involved in the initial defense against infective agents. A major component of innate immunity is the macrophage which performs a range of biological functions, including surveillance, phagocytosis, chemotaxis, and destruction of targeted organisms [19]. Various biological signals activate macrophages, leading to the elimination of numerous harmful substances and pathogens [20]. Nitric oxide (NO) is a mediator of inflammation and is generated by nitric oxide synthase (iNOS) in macrophages [21]. Initiation of a signaling pathway in the macrophage occurs when the bacterial cell wall component (lipopolysaccharide (LPS)) binds to CD14, which is a toll-like receptor (TLR) that triggers a kinase complex cascade. This ultimately leads to the activation and expression of iNOS [22]. Macrophages release inflammatory mediators, such as cytokines. As such, a promising target to enhance immunity could involve the activation of macrophages. Recently, there has been considerable interest in natural polysaccharides because they have exhibited protective effects in humans by strengthening the macrophage-mediated innate immune system [23,24,25].

Extrusion cooking involves the application of high-temperature short time (HTST) and high shear force processes. It has several potential applications in the food industry, particularly in the production of cereals (cooking, formation, and expansion), and for texturizing proteins [26]. The extrusion process consists of several complex reactions, such as gelatinization, thermal treatment, protein denaturation, hydrolysis of protein, mixing, shearing, grinding, shaping, hydration, expanding, partial dehydration, texture alteration, as well as the destruction of pathogens and toxic compounds [27]. Extrusion can be used to pretreat rice straw and speed up the saccharification of rice straw by enzymatic hydrolysis [28]. It has been used to pretreat fish scales and to enhance gelatin extraction from fish scales by hot water [26]. In the current study, we extracted fucoidans from *Sargassum crispifolium* which had been pre-treated by extrusion. The extracted fucoidans were subjected to degradation using ascorbic acid and hydrogen peroxide, which yielded degraded fucoidans with lower molecular weights. These LMW fucoidans were then analyzed to determine their composition and structure, as well as to compare their antioxidant and immunostimulant activities. These results may be of value in further studies aimed at exploring the potential of fucoidans for use in functional foods, cosmetics, and pharmaceuticals.

## 2. Materials and Methods

### 2.1. Materials

Samples of *S. crispifolium* were purchased from a company in southern Taiwan. After washing and drying, the samples were sealed in aluminum foil bags and kept at 4 °C until use. l-fucose, d-galactose, d-glucuronic acid, d-xylose, d-mannose, d-glucose, d-rhamnose, dimethyl sulfoxide (DMSO), potassium bromide (KBr), 2,2,2-Trifluoroacetic acid (TFA), and 3-(4,5-dimethylthiazol-2-yl)-2,5-diphenyltetrazolium bromide (MTT), DPPH, ABTS, and 2,4,6-tripyridyl-s-triazine (TPTZ) were obtained from Sigma-Aldrich (Sigma-Aldrich, St. Louis, MO, USA). DMEM medium, trypsin/EDTA, fetal bovine serum (FBS), penicillin, and streptomycin were purchased from Gibco Laboratories (Gibco Laboratories, Grand Island, NY, USA). Unless otherwise stated, the other reagents were acquired from Sigma-Aldrich.

### 2.2. Compositional Analysis

The crude protein, fat, moisture, and ash were determined according to the AOAC (1990) methods [29]. After drying the samples at 103 °C for 8 h, their moisture content (%) was determined. The crude fat content (%) of the samples was determined gravimetrically using Soxhlet extraction with petroleum ether. A muffle furnace was used to incinerate the sample at 580 °C for 8 h before determining its crude ash content (%), and the crude protein content (N × 5.95) (%) was determined using the Kjeldahl method of acid digested samples.

### 2.3. Extrusion Method

With a slight modification in the method of Wu et al. [9], the extrusion was carried out. Briefly, a single-screw (74 mm diameter) lab extruder (Tsung Hsing Co., Ltd., Kaohsiung, Taiwan) was equipped with a 3.07:1 L/D ratio, and one rounded die head (5 mm diameter) was used. The *S. crispifolium* raw material samples were preconditioned to a 35% moisture level. A constant feed rate of 10.4 kg h^−1^ was used for extrusion. In addition, the barrel temperature and screw speed were set to 115 °C and 360 rpm, respectively. The extrudate of *S. crispifolium* was dried at 55 °C for 30 min and subsequently cooled to reach room temperature (RT). A 20-mesh screen was used to sieve the ground sample and subsequently stored at 4 °C for extraction.

### 2.4. Polysaccharide Extraction Method

According to the formerly described methods [1,5], native fucoidan was extracted from *S. crispifolium*. Briefly, after mixing with 95% ethanol (1:10 *w*/*v*), the algal biomass was shaken at room temperature for 1 h to remove lipids and pigments and then centrifuged at 970× *g* for 10 min. In the next step, the residue was collected and mixed with double distilled water (1:10 *w*/*v*), and the mixture was incubated at 85 °C for 1 h under shaking to extract the algal polysaccharides. The supernatant was collected after centrifuging the mixture at 3870× *g* for 10 min. To precipitate alginate, 2% (*w*/*v*) CaCl_2_ was added to the algal supernatant and held for shaking 1 h at 120 rpm. The supernatant was collected after centrifuging the mixture at 3870× *g* for 10 min. The supernatant was diluted with ethanol (95%) to obtain a final ethanol concentration of 20% for alginic acid precipitation. Fucoidan precipitate was obtained by centrifuging the mixture at 9170× *g* for 30 min, collecting the supernatant, and adding 95% ethanol until a final concentration of 50% ethanol was achieved. Fucoidan obtained by ethanol precipitation was removed by centrifugation at 9170× *g* for 30 min, lyophilized, and stored at 4 °C for further use. The following equation was used to determine the extraction yield.

Extraction yield (%) = (weight of the extracted solid, dry basis/weight of the sample, dry basis) × 100(1)

### 2.5. Degraded Fucoidan Preparation

A 0.1 g of native fucoidan was dissolved in 10 mL of distilled water, and then 10 mmol/L of ascorbic acid or 10 mmol/L of H_2_O_2_ was added. Later, the degradation reaction was initiated by stirring the solution for 1 h. Once the reaction was complete, the digested fucoidan was precipitated with ethanol (75%). The obtained fucoidan was then centrifuged at 9170× *g* for 30 min before lyophilizing.

### 2.6. Analytical Methods

A phenol-sulfuric acid method was used to determine the total sugar content. The reduced sugar content was estimated as per the method described by Yang et al. [4] in which l-fucose was used as a standard. The sulfate content of the sample was measured by hydrolyzing it with a 1 N HCl solution at 105 °C for 5 h. The ion chromatographic analysis of the sample was carried out with a Dionex ICS-1500 using an IonPac AS9-HC column at 1 mL min^−1^ flow rate at 30 °C with conductometric detection to calculate the percentage of sulfate in the sample. K_2_SO_4_ was used as the standard, and 9 mM Na_2_CO_3_ was used as the eluent. BSA was used as a standard for quantifying the protein in the extract by the Bradford method. d-galacturonic acid was used as a standard for the colorimetric determination of uronic acids [30]. Using Folin–Ciocalteu as an analytical method, polyphenols were analyzed using gallic acid as a standard.

### 2.7. HPLC Analysis of Monosaccharide Composition

As a preliminary step towards analyzing monosaccharide composition, the sample was hydrolyzed with 2 M TFA for 4 h at 110 °C. Standard sugars and samples were then derivatized with 1-phenyl-3-methyl-5-pyrazolone (PMP) for 100 min at 70 °C after removing the residual acid. The resulting solution was then extracted trice with chloroform. The separation of monosaccharides was performed using a SHIMADZU HPLC system (Shimadzu, Kyoto, Japan) equipped with a UV-vis detector. On a reversed-phase Inspire^TM^ C18 (250 × 4.6 mm, 5 μm) column (Dikma Technologies, Lake Forest, CA, USA) with detection at 245 nm, the PMP derivatives were eluted with 0.1 M phosphate buffer (pH 6.7) and acetonitrile at a ratio of 83:17 (*v*/*v*, %) at a flow rate of 1 mL/min at 25 °C. The injection volume of all samples was 20 μL. Standards used were d-glucuronic acid, l-fucose, d-galactose, d-mannose, d-xylose, d-glucose, and d-rhamnose.

### 2.8. Molecular Weight Determination

A size-exclusion HPLC column Superdex 200 (300 mm × 10 mm ID) (GE Healthcare, Piscataway, NJ, USA) was used alongside a Shimadzu HPLC system (Shimadzu, Kyoto, Japan) equipped with a refractive index detector to determine the average molecular weights of fucoidans. Chromatography parameters were: eluent 0.2 M NaCl; flow rate 0.4 mL min^−1^; injection vol. 0.15 mL; sample concentration 10 mg mL^−1^; and temperature 25 °C. The HPLC column was calibrated using dextrans of varying molecular weights (5, 50, 150, and 670 kDa).

### 2.9. Fourier Transform Infrared (FTIR) Spectroscopy

FTIR spectra were obtained using the procedure defined by Huang et al. [26]. In short, the sample was evenly ground with KBr (1:50, *w*/*w*) until it reached a size of less than 2.5 microns. Under vacuum at 500 kg/cm^2^, transparent KBr pellets were obtained. Subsequently, the FTIR spectra from the FT-730 spectrometer (Horiba, Kyoto, Japan) were examined. Using 60 scans spanning a range of 4000–400 cm^−1^ at a 16 cm^−1^ resolution, an automatic spectrum collection has been performed, and the collected results have been compared with the KBr background spectrum alone.

### 2.10. Nuclear Magnetic Resonance (NMR) Spectroscopy

To obtain the NMR spectra, the fucoidan sample was first dissolved in 99.9% D_2_O using an NMR tube and then NMR was performed in a Varian VNMRS-700 NMR spectrometer (Varian, Lexington, MA, USA) to obtain spectra data.

### 2.11. Cell Culture

Murine macrophage cell lines RAW 264.7 (BCRC No. 60001) were acquired from the Biosource Collection and Research Center of the Food Industry Research and Development Institute (FIRDI, Hsinchu, Taiwan). The cells were grown in DMEM supplemented with 10% FBS and a 100 U/mL penicillin–streptomycin solution at 37 °C in a humidified chamber with 5% CO_2_, and the medium was changed every 2–3 days.

### 2.12. Measurement of Nitrite Oxide in Culture Media

RAW 264.7 cells (2 × 10^5^ cells/mL) were seeded in a 96-well flat-bottom plate for 24 h at 37 °C with 5% CO_2_. The culture medium was removed and replaced with a fresh medium containing 1 μg/mL of LPS or tested samples at various concentrations. The nitrite concentration was measured in the culture supernatant after incubation for 24 h. In brief, 50 μL of the cultured supernatants were added to a 96-well plate and 100 μL of Griess reagent was added to each well and allowed to stand for 10 min at room temperature. The absorbance at 540 nm was measured using a PowerWave 340 ELISA reader (Bio-Tek Instruments, Winooski, VT, USA), and the quantification of nitrite was standardized with NaNO_2_ at 0–100 μM concentrations [31].

### 2.13. Evaluation of Cytotoxic Activity

Using the MTT assay, all the samples were tested for their cytotoxic activity. In a moistened condition with 5% CO_2_, cells were cultured for 24 h at 37 °C. The medium was then decanted, and the cells were exposed to different concentrations of tested samples in a serum-free medium. A 24 h treatment was followed by a PBS wash, and the MTT reagent was added (0.1 mg/mL). To dissolve the formazan, DMSO was added and thoroughly mixed by pipetting after 2–3 h of incubation. A PowerWave 340 ELISA reader was used to measure the absorption of the resultant solution at 570 nm. The viability of the cell was represented as an MTT reduction percentage.

### 2.14. Quantitation of Cytokines by ELISA

Cells were cultured in the medium at 37  °C in a humidified atmosphere with 5% CO_2_ for 24 h. After removing the medium, the cells were treated with various concentrations of tested samples in the serum-free medium. After 24 h of treatment, the supernatant was collected. IL-6, IL-1β, IL-10, and TNF-α concentrations in supernatants were measured using ELISA Max kits (BioLegend, San Diego, CA, USA) according to the manufacturer’s protocols.

### 2.15. DPPH Radical Scavenging Activity

A previously defined method of Shiao et al. [32]. was used for the radical scavenging activity of DPPH. Briefly, a 50 µL sample was mixed with 150 µL of fresh 0.1 mM DPPH solution dissolved in methanol. After shaking vigorously for 1 min, the mixture was left in the dark at room temperature for 30 min. ELISA readers equipped with PowerWave 340 ELISA readers were used to measure absorbance for all sample solutions at 517 nm. The radical-scavenging activity was derived with the following equation:

DPPH_radical-scavenging_ (%) = (1 − A_sample_/A_control_) × 100 (2)

where A_sample_ represents the absorbance of methanol containing the DPPH solution with tested samples, and A_control_ is the absorbance of methanol containing the DPPH solution without a sample.

### 2.16. ABTS Radical Cation Scavenging Activity

A previous method of Huang et al. [33] was used to measure the scavenging activity of the samples against ABTS radical cation. By reacting 5 mL of 7 mM ABTS solution with 88 µL of 140 mM potassium persulfate, the ABTS^•+^ solution was prepared. Using 95% ethanol, the solution was diluted until an absorbance of 0.70 ± 0.05 at 734 nm was reached. A 100 µL diluted ABTS^•+^ solution was mixed with 100 µL of different sample solutions to perform the assays. An ELISA reader equipped with the PowerWave 340 ELISA reader was used to measure the absorbance at 734 nm after the mixture was reacted for 6 min at room temperature. The blank was prepared in the same manner with distilled water instead of the sample. The activity of scavenging ABTS ^+^ was calculated as follows:

ABTS_cation radical-scavenging_ (%) = (1 − A_sample_/A_control_) × 100 (3)

where A_sample_ represents the ABTS absorbance with tested samples, and A_control_ is the ABTS absorbance without a sample.

### 2.17. FRAP Assay

The FRAP assay was conducted according to the method described by Huang et al. [1]. To prepare the FRAP solution, 10 mL of 300 mM acetate buffer was added with acetic acid until a pH of 3.6 was obtained. This was then dissolved in distilled water with 1 mL of 20 mM ferric chloride hexahydrate and later mixed with 1 mL of 10 mM TPTZ dissolved in 40 mM HCl. Many samples were prepared with 50 μL of the tested sample dissolved at various concentrations. A 450 μL of freshly prepared and warmed (at 37 °C) FRAP solution was added to the sample before reacting in the dark for 30 min, using an equal volume of acetate buffer as a blank. An ELISA reader powered by the PowerWave 340 measured absorbance at 593 nm. The method used to estimate FRAP values was meant to use vitamin C as a standard and to express FRAP values as μmol of vitamin C per gram of dry sample (μmol vitamin C/g dry sample).

### 2.18. Statistical Analysis

All the data are presented as the mean ± SD of three replicates (n = 3). Comparative analysis of values between groups was conducted using the ANOVA as well as by Duncan’s multiple range test. Statistics were considered significant if the *p* value was less than 0.05 (*p* < 0.05).

## 3. Results

### 3.1. Proximate Composition of Sargassum crispifolium

The proximate compositions of *S. crispifolium* (dry weight) are shown in Table 1. *S. crispifolium* consisted of 8.21% ± 0.24% crude protein, 1.17% ± 0.03% crude lipid, 10.3% ± 0.2% ash, and 80.3% ± 0.3% carbohydrate. The major component in *S. crispifolium* was carbohydrate, followed by ash and protein, and the smallest component was lipid.

### 3.2. Preparation of Degraded Fucoidans (ExAh and ExHp) from Extrusion-Pretreated S. crispifolium

In the present investigation, extrusion cooking was applied to pretreat *S. crispifolium*. Crude fucoidan was then extracted from non-extruded and extrusion-pretreated *S. crispifolium*. Table 2 shows the extrusion parameters and water extraction conditions that were used. The extraction yields of fucoidan for NEx and Ex were 1.50 ± 0.19 and 2.59 ± 0.32 g/100 g (dry basis), respectively (Table 2). These results reveal that the extrusion-pretreatment process resulted in a 1.73-fold (2.59/1.50 = 1.73) increase in the extraction yield of fucoidan, which was statistically significant. Ex was used for the degradation experiments. Two degradation reagents, ascorbic acid, and hydrogen peroxide were utilized to degrade Ex. Two degraded fucoidans—namely, ExAh (degraded by ascorbic acid) and ExHp (degraded by hydrogen peroxide)—were obtained. High-performance liquid chromatography (HPLC) gel filtration analysis was then conducted to determine the molecular weights (MWs) and molecular weight distributions of fucoidans and thereby establish whether or not the reagents had successfully degraded Ex. Table 3 shows that the average molecular weights of the extracts for Ex were 50.8 kDa (peak area = 70.3%), 2.34 kDa (peak area = 28.6%), and 0.90 kDa (peak area = 1.13%); for ExAh, they were 50.7 kDa (peak area = 64.7%), 2.36 kDa (peak area = 33.7%), and 0.90 kDa (peak area = 1.58%); and for ExHp, they were 2.28 kDa (peak area = 45.1%) and 0.88 kDa (peak area = 54.9%). These data suggest that ExHp had the smallest molecular weight, followed by ExAh, and then Ex.

### 3.3. Compositional and Structural Analyses of Native and Degraded Fucoidans

**Figure 1:**
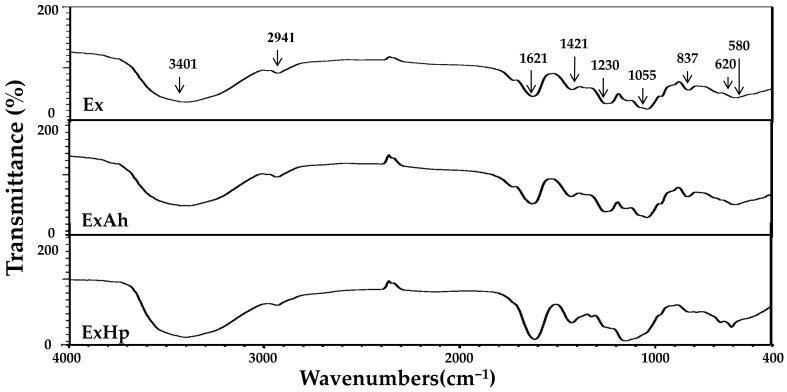
FTIR spectra for Ex, ExAh, and ExHp. Absorption bands at 3401, 2941, 1621, 1421, 1230, 1055, 837, 620, and 580 cm^−1^ are indicated.

**Figure 2:**
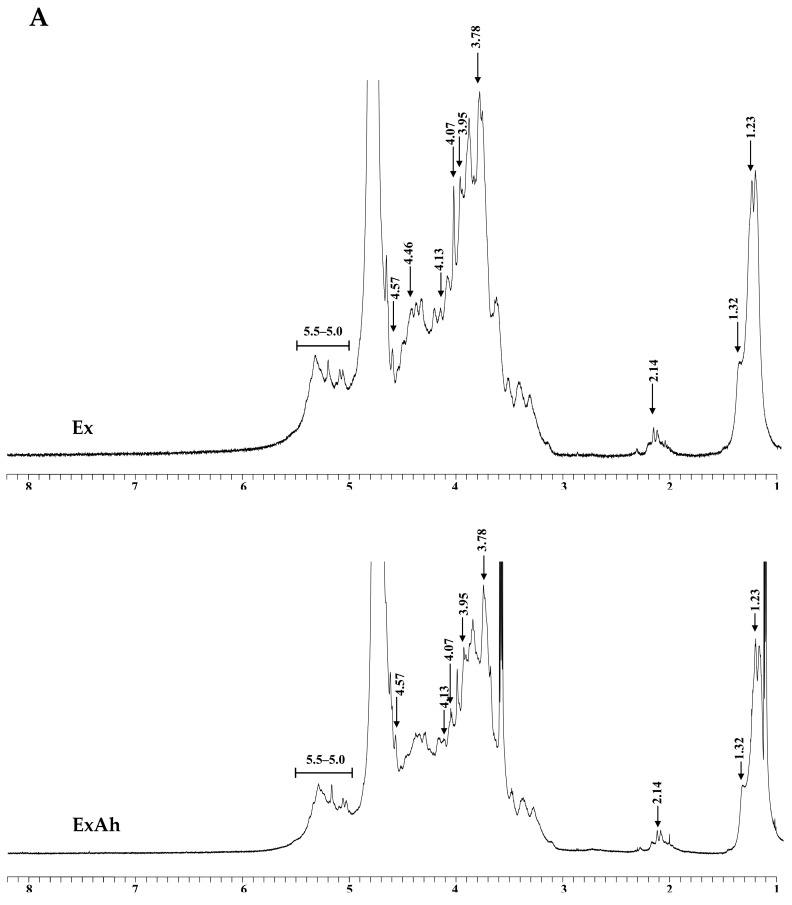

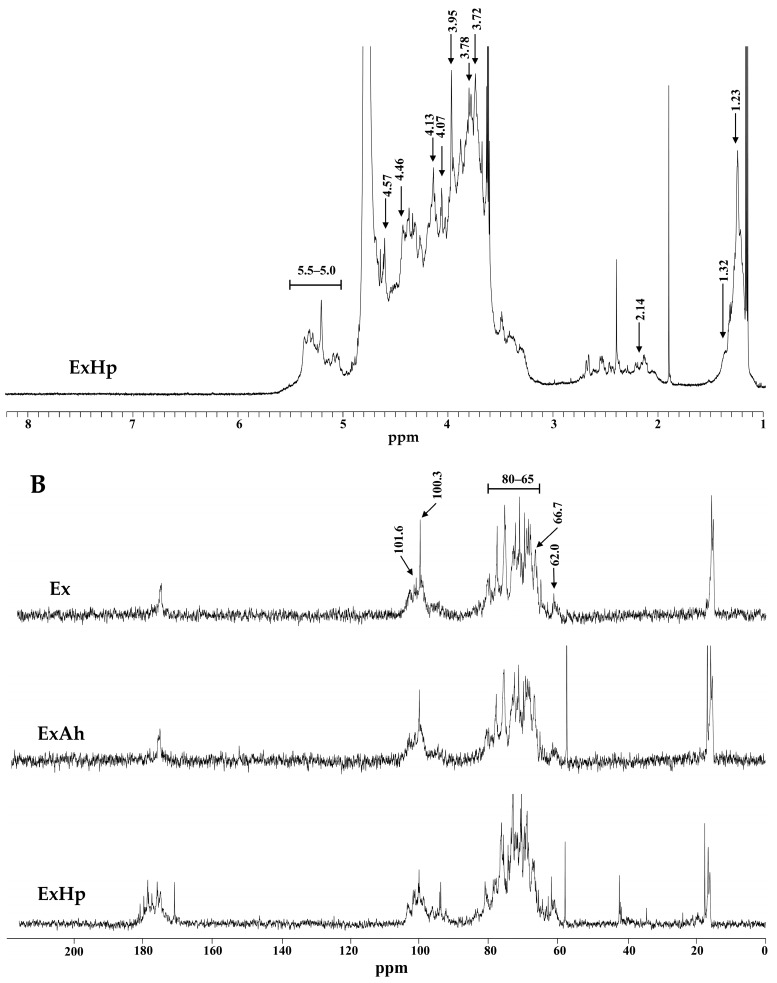
NMR spectra for Ex, ExAh, and ExHp. (**A**) ^1^H-NMR of Ex, ExAh, and ExHp and (**B**) ^13^C-NMR of Ex, ExAh, and ExHp. The characteristic peaks are indicated in each graph.

Total sugar, fucose, sulfate, uronic acid, protein, and polyphenols contents, as well as the monosaccharide composition of Ex, ExAh, and ExHp, are displayed in Table 4. The total sugar contents of Ex, ExAh, and ExHp were 57.5% ± 2.3%, 53.8% ± 2.6%, and 39.1% ± 1.4%, respectively. The degradation of fucoidan led to lower total sugar content. The fucose contents of Ex, ExAh, and ExHp were 48.8% ± 2.1%, 45.8% ± 1.2%, and 40.1% ± 2.0%, respectively. The fucose content decreased after subjecting the fucoidan to degradation treatment. The sulfate content percentages of Ex, ExAh, and ExHp were 32.9% ± 0.4%, 22.4% ± 0.8%, and 26.9% ± 0.5%, respectively. Overall, the sulfate content of polysaccharides was lower after degradation treatment. These results indicate that the degradation of fucoidan decreased its total sugar, fucose, and sulfate contents. Here, in Table 4, the uronic acid content percentages of Ex, ExAh, and ExHp were 16.7% ± 0.7%, 17.9% ± 0.1%, and 13.6% ± 0.4%, respectively. The protein content percentages of Ex, ExAh, and ExHp were 0.58% ± 0.02%, 0.69% ± 0.06%, and 0.42% ± 0.02%, respectively. Moreover, the polyphenol content percentages of Ex, ExAh, and ExHp were 0.20% ± 0.00%, 0.43% ± 0.01%, and 0.78% ± 0.01%, respectively. Basically, degradation of Ex increased polyphenol content. In Table 4, the monosaccharide compositions of Ex, ExAh, and ExHp can be seen. Fucose, galactose, and mannose were the most prevalent sugar units, and other monosaccharides, including glucuronic acid, glucose, rhamnose, and xylose were also detected in the polysaccharide samples. ExHp showed greater amounts of galactose, mannose, glucuronic acid, and glucose compared with those of Ex and ExAh. Furthermore, the molar ratios of monosaccharides did not significantly differ between Ex and ExAh. FTIR and NMR techniques were used to perform structural analyses of Ex, ExAh, and ExHp. As depicted in Figure 1, the IR bands at 3401 and 2941 cm^−1^ correspond to the stretching vibrations of OH and H_2_O and the stretching vibrations of C–H in the pyranoid ring or C-6 in the fucose and galactose groups [34,35]. There were absorption bands observed at 1621 and 1421 cm^−1^, suggesting H_2_O scission and in-plane ring vibrations of COH, CCH, and OCH, which are known polysaccharide absorption patterns [34,35,36]. As evidenced by peaks at 1230 and 1055 cm^−1^, these peak positions are due to asymmetrical stretching vibrations of the S=O and the C–O–C stretching vibrations in the ring or C–O–H of the glycosidic bond [34,35]. An explanation for the absorption bands at 837 cm^−1^ can be found in the equatorial C–O–S bending vibration of the sulfate substituent at the axial position C-4 [1]. A symmetrical and anti-symmetrical O=S=O deformation can be seen at 620 and 580 cm^−1^, respectively [37]. Figure 2A depicts the ^1^H-NMR spectra of Ex, ExAh, and ExHp. The signals in the range 5.5 to 5.0 ppm are due to l-fucopyranosyl units [38]. The signals at 4.57 and 4.46 ppm, most notably in Ex, indicate the presence of H-2 in a 2-sulfated fucopyranose residue [38], and the signal at 4.13 ppm (4[H]) is suggestive of the presence of 3-linked α-l-fucose [38]. Signals with a ppm of 4.07/3.95 (6[H]/6′[H]) denote the presence of a (1–6)-β-d-linked galactan [39]. The signals detected at 3.78 ppm and 3.72 ppm may be indicative of the existence of (3[H]) 4-linked β-d-galactose and (4[H]) 2,3-linked α-β-mannose, respectively [36]. The signals between 2.20 and 2.14 ppm may indicate methyl protons in *O*-acetyls [36,40], which are frequently detected in algal polysaccharides. In addition, the signal at 1.32 ppm indicates the C6 methyl protons of l-fucopyranose [41]. The signal at 1.23 ppm (6[H]) demonstrates an alkane proton in two methyl groups [42]. Figure 2B depicts the ^13^C-NMR spectra of Ex, ExAh, and ExHp. As with numerous other algal fucoidans [41,43], our extracted fucoidans exhibited a highly complex ^13^C NMR spectrum and thus a complete interpretation could not be performed. Figure 2B shows that Ex contains a few intense signals in the anomeric (97–102 ppm) and high-field (16.5–16.7 ppm) regions, which typically denote the presence of α-fucopyranosides [43]. Furthermore, the signals from 105 to 74 ppm appear to correspond to sulfated or glycosylated carbons in the fucopyranose ring, and the cluster of signals from 71 to 66 ppm may be explained by unsubstituted carbons [44]. The prominent signal at 101.6 ppm and the peaks from 80 to 65 ppm suggest the presence of (1–6)-β-d-linked galactan [39]. The signal at 100.3 ppm is suggestive of (1,3)-linked α-l-fucopyranose residue [45]. The signals at 66.7 and 62.0 ppm indicate the existence of β-d-galactopyranose residues [46]. In summary, the FTIR, ^1^H-NMR, and ^13^C-NMR experiments demonstrate that Ex, ExAh, and ExHp possess the structural features that are characteristic of fucose-containing sulfated polysaccharides.

### 3.4. Antioxidant Activity of Native and Degraded Fucoidans

To determine the antioxidant activity of Ex, ExAh, and ExHp in the present study, the following methods were adopted: 2,2′-azino-bis(3-ethylbenzothiazoline-6-sulphonic acid) diammonium salt (ABTS), 2,2-diphenyl-1-picrylhydrazyl (DPPH), and ferric reducing antioxidant power (FRAP) analyses. The results shown in Table 5 indicate that the IC_50_ of DPPH radical scavenging activity for Ex, ExAh, and ExHp were 5.67 ± 0.05, 5.34 ± 0.27, and 3.67 ± 0.15 (mg/mL), respectively. The degradation of polysaccharides led to increased DPPH radical scavenging activity. The IC_50_ of ABTS^•+^ scavenging activity for Ex, ExAh, and ExHp were 0.97 ± 0.01, 0.93 ± 0.02, and 0.32 ± 0.01 (mg/mL), respectively. Similar to that of DPPH radical scavenging activity, the degradation of polysaccharides led to augmented ABTS^•+^ scavenging activity. The FRAP values of the Ex, ExAh, and ExHp were 2.33 ± 0.04, 3.52 ± 0.12, and 6.15 ± 0.09 (μmol vitamin c/g extract, dry basis), respectively. The degradation of polysaccharides significantly enhanced the antioxidant activity as illustrated by FRAP assay. In summary, these results indicate that the reduction in molecular weight of polysaccharides possibly increased its antioxidant activities.

### 3.5. Activation of Macrophage Cytokine Release by Native and Degraded Fucoidans

**Figure 3:**
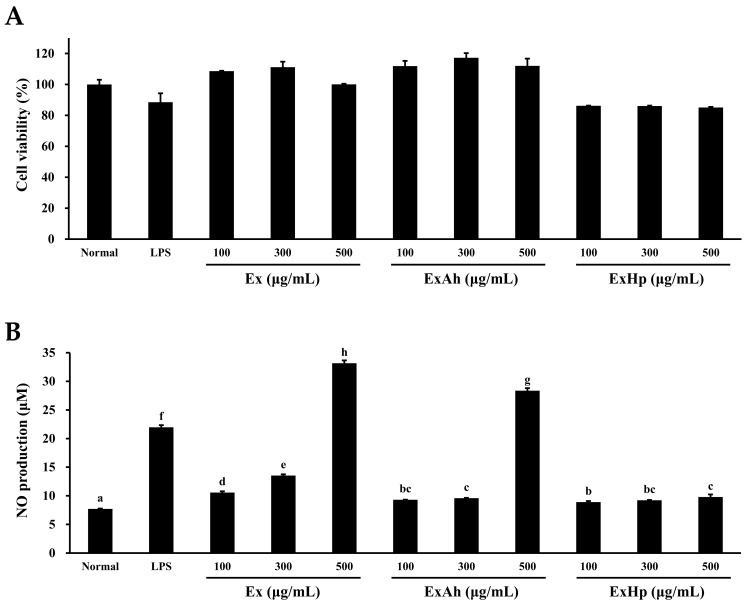

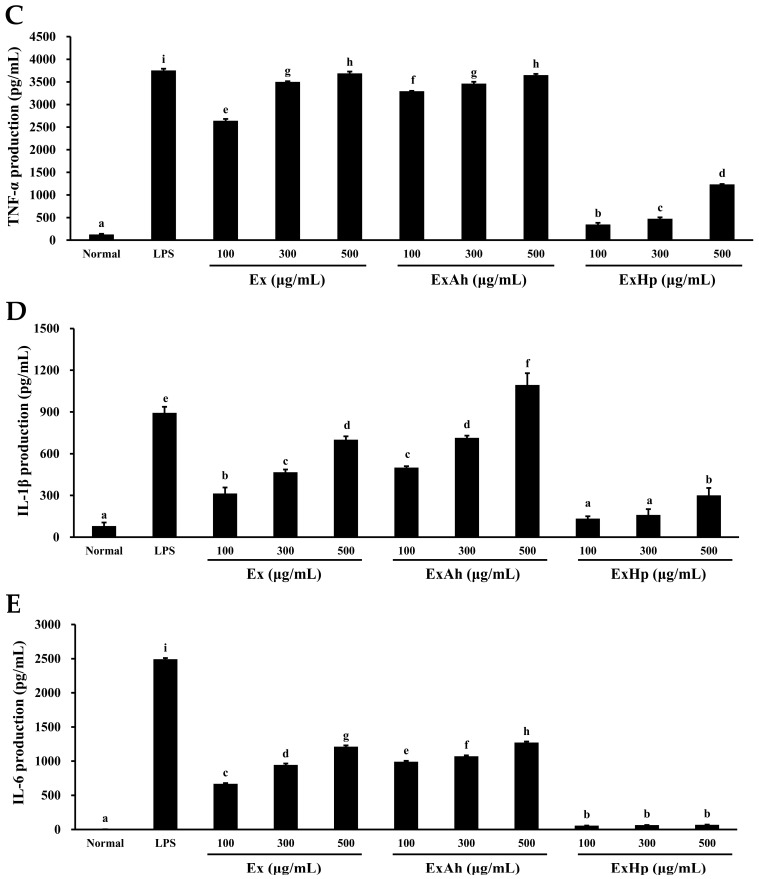

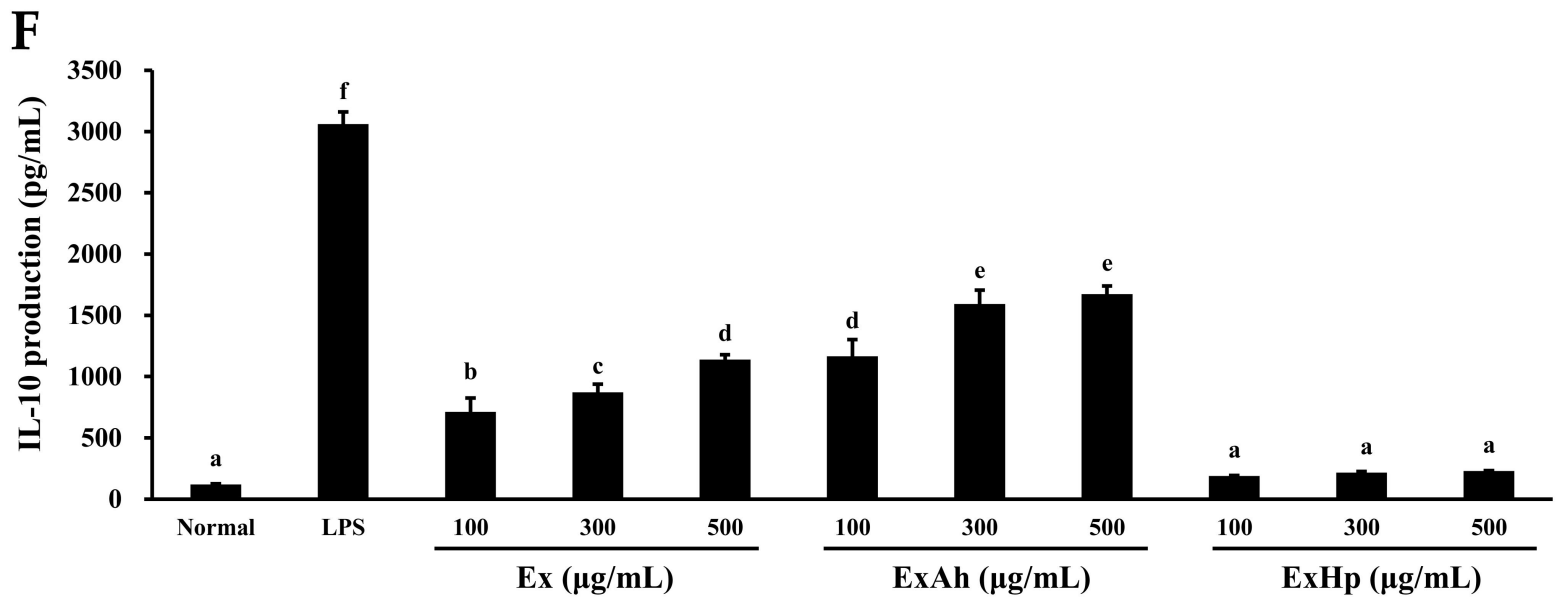
Effects of various concentrations of Ex, ExAh, and ExHp on cell viability, NO production, TNF-α production, IL-1β production, IL-6 production, and IL-10 production in RAW 264.7 macrophages. (**A**) cell viability, (**B**) NO production, (**C**) TNF-α production, (**D**) IL-1β production, (**E**) IL-6 production, and (**F**) IL-10 production. Values are mean ± SD (n = 3); values in the same graph with different letters (in a, b, c, d, e, f, g, h, and i) are significantly different (*p* < 0.05).

The effects of Ex, ExAh, and ExHp on macrophage function were investigated using RAW 264.7 cells. First, the cell viability of RAW 264.7 cells was analyzed following exposure to Ex, ExAh, and ExHp. In Figure 3A, the cell viabilities of RAW 264.7 cells were 85.1% ± 0.3% to 117.2% ± 3.1%. The results suggest that there were no marked alterations in the cell viabilities of RAW 264.7 cells following LPS and polysaccharide treatments at different concentrations. NO production levels can serve as a quantitative index of macrophage activation. Figure 3B shows that the basal level of NO production in untreated cells was low. LPS was used as a positive control and caused a significant increase in the production of NO in RAW 264.7 cells. Furthermore, NO production in RAW 264.7 cells was significantly increased by Ex dose-dependently. ExAh also induced a significant enhancement of NO production in RAW 264.7 cells. However, ExHp only caused a slight enhancement of NO production in RAW 264.7 cells. The effects of Ex, ExAh, and ExHp on inflammatory cytokines (TNF-α, IL-1β, IL-6, and IL-10) were also evaluated. LPS induced a significant increase in the production of TNF-α in RAW 264.7 cells (Figure 3C). There was a significant dose-dependent increase in TNF-α production in RAW 264.7 cells following treatment with Ex, ExAh, and ExHp. In addition, ExAh tended to have a stronger effect on the promotion of TNF-α production in RAW 264.7 cells compared with that achieved by Ex and ExHp. In Figure 3D, it can be seen that LPS significantly enhanced IL-1β production in RAW 264.7 cells. Ex, ExAh, and ExHp caused a dose-dependent and significant increase in IL-1β production in RAW 264.7 cells. ExAh showed the largest inductive effect on IL-1β production in RAW 264.7 cells among Ex, ExAh, and ExHp. LPS significantly elevated IL-6 production in RAW 264.7 cells, as shown in Figure 3E. Ex and ExAh induced a significant dose-dependent increase in IL-6 production in RAW 264.7 cells. In general, ExAh was more capable of inducing an increase in IL-6 production in RAW 264.7 cells in comparison with Ex and ExHp. Figure 3F shows that LPS significantly elevated IL-10 production in RAW 264.7 cells. Ex and ExAh induced a significant dose-dependent increase in IL-10 production in RAW 264.7 cells. Generally, ExAh was more capable of inducing an increase in IL-10 production in RAW 264.7 cells in comparison with Ex and ExHp. In summary, ExAh was more capable of elevating production levels of NO, TNF-α, IL-1β, IL-6, and IL-10 in RAW 264.7 cells as compared to Ex and ExHp. However, ExHp could only negligibly or slightly induce the production of NO, TNF-α, IL-1β, IL-6, and IL-10 in RAW 264.7 cells.

## 4. Discussion

The sample of *S. crispifolium* used in the present study consisted of 8.21% protein, 1.17% lipid, 10.3% ash, and 80.3% carbohydrate (dry basis) (Table 1). Other investigations reported that the chemical composition of *S. crassifolium* was found to be 2.36% protein, 0.98% lipid, 34.0% ash, and 62.7% carbohydrate (dry basis) [4]; the chemical composition of *S. glaucescens* was found to be 2.51% protein, 1.04% lipid, 37.0% ash, and 60.0% carbohydrate (dry basis) [5]; the chemical composition of *S. cristaefolium* was found to be 3.83% protein, 1.51% lipid, 24.8% ash, and 69.9% carbohydrate (dry basis) [5]; the chemical composition of *S. horneri* was found to be 10.6% protein, 0.99% lipid, 26.7% ash, and 61.8% carbohydrate (dry basis) [47]. In general, *S. crispifolium* contained the highest amount of carbohydrate as compared to other *Sargassum* spp., suggesting *S. crispifolium* is a good source for extracting fucoidan.

Extrusion cooking provides several benefits, including ease of operation, continuous production, high production yield, low manpower, minimal waste, and diverse products [26]. Several investigations have demonstrated that extrusion can be successfully applied in the pretreatment of rice straw, which involves enhancing the saccharification of rice straw by enzymatic hydrolysis [28]. Pretreatment of soybean dregs by extrusion reduces the quantity of insoluble dietary fiber (IDF) and boosts the level of soluble dietary fiber (SDF) in soybean residues [48]. Furthermore, the extrusion process can be used to increase the extraction yield of *S. crassifolium* fucoidan (compared with a non-extruded sample) [9]. In the present study, we pretreated *S. crispifolium* by single-screw extrusion to increase the extraction yield of fucoidan. Table 2 shows that the extraction yield of fucoidan for Ex was significantly greater than that of NEx, which demonstrates that the extrusion-pretreatment did indeed increase the extraction yield of fucoidan.

The biological functions of fucoidans are governed by their molecular structures, such as their fucose linkage, type of sugar, sulfate content, and molecular weight. The molecular weight of polysaccharides appears to be the most important factor determining their biological properties [49]. High molecular weight polysaccharides suffer from low solubility and cannot be readily processed, which limits their ability to penetrate the cell. Conversely, fucoidans with lower molecular weight tend to exhibit higher biological functions, which include anticancer, anticoagulation, and antioxidant activities [8,10]. In the present investigation, two degradation reagents—namely, ascorbic acid and hydrogen peroxide—were used for the degradation of Ex. Two degraded products were obtained, as follows: ExAh and ExHp. Among them, ExHp had the lowest molecular weight (Table 3). The results in Table 4 show that ExHp had the lowest total sugar and fucose contents compared with those of Ex and ExAh. These results are in line with previously published findings [50], which indicate that the fall in total sugar and fucose contents of fucoidan paralleled the decrease in the molecular weight of fucoidan after the degradation treatment. FTIR and NMR analyses were conducted to determine the structure of fucoidans. In Figure 1, it can be seen that Ex, ExAh, and ExHp had a similar FTIR profile, which indicates that the structural components of polysaccharides and the sulfate group positions were not markedly changed by the degradation treatments. This observation is consistent with previous results [3]. In addition, the FTIR and NMR experiments showed that Ex, ExAh, and ExHp possess the structural features that are characteristic of fucose-containing sulfated polysaccharides, which is similar to previous investigations [1].

There are data in the literature that suggest LMW fucoidans possess greater antioxidant activities compared with their native fucoidan [3]. A similar finding was obtained in the current study. Table 3 shows that ExHp had the smallest molecular weight, followed by ExAh, and then Ex. Moreover, ExHp showed the greatest DPPH, ABTS, and FRAP antioxidant activities, followed by ExAh, and then Ex (Table 5). Studies suggested that crude extract of fucoidan may contain impurities such as polyphenols and it may be hard to get rid of them [2]. In Table 4, we also found that ExHp had the greatest number of polyphenols, followed by ExAh, and then Ex, which is in parallel with the finding that ExHp had the greatest antioxidant activities, followed by ExAh, and then Ex. Although the number of polyphenols coextracted is tiny, it cannot be excluded that the coextracted polyphenols may also have contributions to the antioxidant activities. In summary, ExHp possessed the greatest antioxidant activities among the fucoidan extracted, which may correlate to the low molecular weight and high polyphenol content in it.

NO is an inflammatory mediator whose action is triggered by the presence of inflammatory cytokines or bacterial LPS in several cell types, such as macrophages [51]. Figure 3B shows that LPS significantly enhanced the production of NO in RAW 264.7 macrophages compared with the control. Ex and ExAh caused a dose-dependent increase in NO production in RAW 264.7 macrophages. ExHp was also capable of inducing NO production, but the fold increase in NO was notably lower compared with those induced by Ex and ExAh. Fucoidan was shown to significantly reduce the production of inflammatory mediators and cytokines in LPS-stimulated RAW 264.7 macrophages by reducing their gene expression [52]. Fucoidan exhibited anti-inflammatory properties by blocking LPS-induced nuclear translocation of NF-κB and the accumulation of ROS. However, other investigations reported enhanced production of pro-inflammatory cytokines by fucoidan [53]. For example, the effect of *Nizamuddinia zanardinii* fucoidan on RAW 264.7 murine macrophages and NK-92 cells was examined by Tabarsa et al. [54]. A fraction of an extract from the brown seaweed exhibited significant immune-enhancing activity and enhanced NO, TNF-α, IL-1β, and IL-6 production, moreover, activating NF-κB, NK cells, and signaling pathways of MAPK, with successive production of INF-γ and TNF-α. Further investigations have shown that fucoidan derived from *Ascophyllum nodosum* and *Fucus vesiculosus* induce NO and cytokine synthesis in RAW 264.7 cells by activating signaling pathways of NF-κB and AP-1 [55]. Hence, taken together, the aforementioned findings indicate that the immunostimulant activity of fucoidan might be related to specific activation of TLR-2 or TLR-4 and subsequent activation of NF-κB or MAPKs pathways and increased production of IL-6, IL-8, IL-1β, NO, TNF-α, and INF-γ, depending on the brown algae species being studied [53]. In Figure 3, the immune mediators TNF-α (a pro-inflammatory cytokine), IL-1β (a pro-inflammatory cytokine), IL-6 (a pro-inflammatory cytokine), and IL-10 (an anti-inflammatory cytokine) displayed a trend, i.e., ExAh induced the highest fold increase as compared with Ex and ExHp. Makarenkova et al. [56] investigated the interactions of three fucoidans (fucoidan from *Laminaria japonica*, MW = 10–30 kDa; fucoidan from *L. cichorioides*, MW = 40–80 kDa; fucoidan from *Fucus evanescens*, MW = 40–60 kDa) with TLRs, and the results showed that they triggered elevated production of cytokines and chemokines, as well as greater expression of MHC molecules. The MW of these fucoidans was approximately 30–80 kDa. In the current study, the MW distribution of Ex was as follows: 50.8 kDa (peak area = 70.3%), 2.34 kDa (peak area = 28.6%), and 0.90 kDa (peak area = 1.13%). The MW distribution of ExAh was 50.7 kDa (peak area = 64.7%), 2.36 kDa (peak area = 33.7%), and 0.90 kDa (peak area = 1.58%). The MW distribution of ExHp was 2.28 kDa (peak area = 45.1%) and 0.88 kDa (peak area = 54.9%) (Table 3). It can therefore be inferred that Ex and ExAh possess a more suitable MW than ExHp for stimulating immune activity. In summary, Ex and ExAh exhibited higher immunostimulant activity compared with ExHp, possibly due to the approximate MW in Ex and ExAh. However, more research is needed to further explore these issues.

In conclusion, *S. crispifolium* was pretreated by single-screw extrusion to efficiently extract native fucoidan (Ex). Comparing the extruded sample with the non-extruded sample, the extrusion pretreatment process enhanced fucoidan extraction yield. Both degraded fucoidans, ExAh and ExHp, were made by degrading Ex with ascorbic acid and H_2_O_2_, respectively. Analyses of Ex, ExAh, and ExHp disclosed differences in the molecular weight and chemical compositions but similarities in structural features. A reduction in the molecular weight of fucoidan appeared to enhance antioxidant activities. Immunostimulant analyses revealed that ExAh induced the greatest fold increase in NO, TNF-α, IL-1β, IL-6, and IL-10 among the three fucoidans tested. Thus, ExAh may have the potential as effective immunostimulant agent for industrial usage. Further in vivo research is needed to elucidate the signaling mechanisms involved in ExAh-induced immune responses.

## Figures and Tables

**Table 1:** Proximate composition of *Sargassum crispifolium*.

Proximate Analysis (%)	*S. crispifolium*
Crude protein	8.21 ± 0.24 ^1^
Crude lipid	1.17 ± 0.03
Ash	10.3 ± 0.2
Carbohydrate	80.3 ± 0.3

^1^ Values are mean ± SD (n = 3).

**Table 2:** Extrusion variables, extraction variables, and extraction yields of crude extracts of fucoidan for NEx and Ex.

Variables of Extrusion	NEx	Ex
Preconditioning solvent	-	ddH_2_O
The moisture content of algal powder (%)	10	35
Feed supply (kg/h)	-	10.4
Die diameter (mm)	-	5
Screw speed (rpm)	-	360
Barrel temperature (°C)	-	115
**Variables of Water Extraction**	**NEx**	**Ex**
Extraction temperature (°C)	85	85
Extraction time (h)	1	1
**Extraction Yield of Fucoidans**	**NEx**	**Ex**
Extraction yield (%, dry basis)	1.50 ± 0.19 ^1^	2.59 ± 0.32 **

-, not adopted. ^1^ Values are mean ± SD (n = 3); **, *p* < 0.01.

**Table 3:** Molecular weight analysis for Ex, ExAh, and ExHp.

Fucoidans	Peak 1 (MW (kDa)/Peak Area (%))	Peak 2 (MW (kDa)/Peak Area (%))	Peak 3 (MW (kDa)/Peak Area (%))
Ex ^1^	50.8/70.3	2.34/28.6	0.90/1.13
ExAh ^1^	50.7/64.7	2.36/33.7	0.90/1.58
ExHp ^1^	ND ^2^	2.28/45.1	0.88/54.9

^1^ Ex (fucoidan from extrusion-pretreated *Sargassum crispifolium*); ExAh (Ex degraded by ascorbic acid); ExHp (Ex degraded by hydrogen peroxide). ^2^ ND: not detected.

**Table 4:** Composition analyses for Ex, ExAh, and ExHp.

Chemical Composition	Ex ^1^	ExAh ^1^	ExHp ^1^
Total sugar (%) ^2^	57.5 ± 2.3 ^b^	53.8 ± 2.6 ^b^	39.1 ± 1.4 ^a^
Fucose (%) ^2^	48.8 ± 2.1 ^b^	45.8 ± 1.2 ^b^	40.1 ± 2.0 ^a^
Sulfate (%) ^2^	32.9 ± 0.4 ^c^	22.4 ± 0.8 ^a^	26.9 ± 0.5 ^b^
Uronic acid (%) ^2^	16.7 ± 0.7 ^b^	17.9 ± 0.1 ^c^	13.6 ± 0.4 ^a^
Protein (%) ^2^	0.58 ± 0.02 ^b^	0.69 ± 0.06 ^c^	0.42 ± 0.02 ^a^
Polyphenols (%) ^2^	0.20 ± 0.00 ^a^	0.43 ± 0.01 ^b^	0.78 ± 0.01 ^c^
**Monosaccharide composition** **(molar ratio)**	**Ex**	**ExAh**	**ExHp**
Fucose	1	1	1
Galactose	0.29	0.29	0.42
Mannose	0.50	0.50	1.17
Glucuronic acid	0.10	0.10	0.21
Glucose	0.08	0.07	0.13
Rhamnose	0.01	0.01	0.01
Xylose	0.15	0.14	0.16

^1^ Ex (fucoidan from extrusion-pretreated *Sargassum crispifolium*); ExAh (Ex degraded by ascorbic acid); ExHp (Ex degraded by hydrogen peroxide). ^2^ Total sugars (%), fucose (%), sulfate (%), uronic acid (%), protein (%), and polyphenols (%) = (g/g_sample, dry basis_) × 100; Values are mean ± SD (n = 3); values in the same row with different letters (in ^a^, ^b^, and ^c^) are significantly different (*p* < 0.05).

**Table 5:** Antioxidant activities of Ex, ExAh, and ExHp.

Treatments	DPPH/IC_50_ (mg/mL) ^1^	ABTS/IC_50_ (mg/mL) ^1^	FRAP/Vitamin C Equivalent (μmol Vitamin c/g Extract, Dry Basis)
Ex	5.67 ± 0.05 ^2,c^	0.97 ± 0.01 ^c^	2.33 ± 0.04 ^a^
ExAh	5.34 ± 0.27 ^b^	0.93 ± 0.02 ^b^	3.52 ± 0.12 ^b^
ExHp	3.67 ± 0.15 ^a^	0.32 ± 0.01 ^a^	6.15 ± 0.09 ^c^
Vitamin C	0.010 ± 0.000	0.003 ± 0.000	-

^1^ IC_50_ value: concentration of fucoidan sample capable of scavenging 50% of DPPH or ABTS free radicals; ^2^ Values are mean ± SD (n = 3); values in the same column with different letters (in ^a^, ^b^, and ^c^) are significantly different (*p* < 0.05).

## Data Availability

Data is contained within the article.
